# Freudarwin: Evolutionary Thinking as a Root of Psychoanalysis

**DOI:** 10.3389/fpsyg.2018.00892

**Published:** 2018-06-19

**Authors:** Geoffrey Marcaggi, Fabian Guénolé

**Affiliations:** ^1^Clinique du Bocage, Louvroil, France; ^2^Service de Psychiatrie de l’Enfant et de l’Adolescent, Centre Hospitalier Universitaire de Caen, Caen, France; ^3^UFR de Médecine, Université Caen Normandie, Caen, France; ^4^INSERM, U1077, Caen, France; ^5^École Pratique des Hautes Études, Paris, France

**Keywords:** psychological adaptation, biological evolution, Charles Darwin, expressed emotion, 20th century history, psychoanalysis, genetic selection

## Abstract

This essay synthesizes the place of biological evolutionism in the early history of psychoanalysis, and shows the implicit significance of German Darwinism in Sigmund Freud’s whole psychoanalytical works. In particular, Freud, together with Sándor Ferenczi (1873–1933), applied to mental disorders hypotheses inspired by August Pauly’s (1850–1914) psychological Lamarckism and Ernst Heckel (1834–1919) theory of recapitulation. Both of these theories rested upon the principle of inheritance of acquired characteristics, and were disproved by biological discoveries during the interwar period. However, despite these scientific progresses, Freud never gave up his idea of inherited unconscious memories, and we try here to sketch out what would have cost him a renunciation to such outdated biological principles. Notwithstanding, Sigmund Freud was the first to elaborate on evolutionary causes of mental syndromes, which makes of him the forerunner of current neo-Darwinian psychopathology, with few continuators to date within the psychoanalytic field. Nowadays, the extended neo-Darwinian synthesis and affective neuroscience may pave the way for a rational Darwinian approach to human mental disorders, which would take into account the whole neurological and psychological evolution of species, and be centered on emotions and their vicissitudes.

## Introduction: From an Evolutionary Point of View...

“Nothing in biology makes sense except in the light of evolution” (Dobzhansky, 1964).

This sentence from famous biologist Theodosius Dobzhansky (1900–1975) expresses the ultimate place which occupies nowadays the neo-Darwinian theory of evolution in the epistemology of the life sciences: any observed biological phenomenon would rely on it in the final analysis (Bowler, 1989). This applies in part to psychology and psychopathology, through the current fields of “evolutionary psychology” (Tooby and Cosmides, 1995), “evolutionary psychopathology” (Baron-Cohen, 1997), and “evolutionary psychiatry” or “Darwinian psychiatry” (Stevens and Price, 1996; McGuire and Troisi, 1998). Though this neo-Darwinian psychopathology appears questionable on many theoretical points (Panksepp and Panksepp, 2000; Marcaggi and Guénolé, 2018), and the clinical practices that it inspires seem marginal, it is nonetheless present under the essential generalities in the most important international journals of psychiatry and psychopathology (Brüne et al., 2012).

Sigmund Freud (1856–1939) is generally considered as the main precursor of current evolutionary psychopathology (Stevens and Price, 1996; McGuire and Troisi, 1998; de Block, 2006), as several of his works involved Darwinian concepts or, more globally, evolutionary ones. Actually, in a famous study covering the relationships between Freud’s works and biology, Sulloway had gone so far as to write that “nowhere was the impact of Darwin, direct and indirect, more exemplary or fruitful outside of biology proper than within Freudian psychoanalysis” (Sulloway, 1979, p. 275).

Sigmund Freud was born just a few years before Charles Darwin (1809–1882) published his *On the origin of species by means of natural selection* (Darwin, 1859), and the whole scientific generation to which he belonged in the German-speaking area was indeed deeply influenced by Darwin’s works and their German appropriation (Gliboff, 2008; Richards, 2013).

This influence of evolutionary thinking of his time on Freud’s works is however not so much obvious at first sight when reading the texts he published during his lifetime – which is probably why this influence has been contested (Holmes, 1983). Indeed, like it was the case in a way for his inspirations from philosophers (Assoun, 1995), evolutionary concepts and their logic are mostly implicit in Freud’s works, who never expressed them in a fully integrated manner. This issue thus finally appears like a puzzle, which can be assembled only in outline and by taking into account not only Freud’s lifetime works, but also his posthumous ones, his correspondence, and other historical material like accounts of people who knew him.

The aim of this historical essay was thus to synthesize in an updated way the major yet little known place of evolutionary thinking in the works of Sigmund Freud. The result is intended as a basis for reflection for all those who will take an interest, clinically and/or theoretically, in the place of evolutionary thinking in the development and future of psychoanalytic psychopathology.

## Evolution

“[...] the view which most naturalists entertain, and which I formerly entertained – namely, that each species has been independently created – is erroneous.” (Darwin, 1859, p. 6)

It would be wrong to attribute to Charles Darwin the whole paternity of biological evolutionism. Indeed, the idea that species were changing with time had already been stated by some ancient philosophers, like Empedocles in the 5th century before our era, or his Chinese contemporary Tson Tse, who additionally postulated a common ancestor of living species (Bowler, 1989). However, it was only at the beginning of the 19th century that the French philosopher and naturalist Jean-Baptiste de Lamarck (1744–1829) developed the first explanatory theory of the transformation of species (Lamarck, 1801). Unjustly, Lamarck is generally held to be concerned only with the idea of hereditary transmission of acquired characteristics that his transformism included: according to Lamarck, individuals were evolving during their life by interacting with their environment, and these modifications were transmitted to the offsprings (Lamarck, 1809) – an idea that became obsolete some decades later^1^, but which everyone approved at this time, including Darwin (Darwin, 1868, 1872). In fact, the real specificity of the Lamarckian theory, from which Darwinian evolutionism will partly stand out, does not concern inheritance of acquired characteristics, but the mechanism supposed to be at work in the transformation of the species.

For Lamarck, repeated actions resulting from needs were producing a progressive physiological transformation of individuals, in the direction of adaptation to the environment (Corsi, 1992). He thus did not take into account what will be one of the fundamental bases of Darwin’s theory of evolution: the intrinsic variability of species (Ruse, 2009). Considering the variability of traits among individuals actually makes it possible to understand the natural selection mechanism of Darwin: within a species, individuals spontaneously exhibit variations of traits, which appear spontaneously and may or may not represent an advantage for survival and reproduction. Certain individuals with beneficial variation will thus be more likely to survive and reproduce: variability induces natural selection within the species. The phenomenon is well-known to growers and breeders, who are accustomed to making an artificial selection for optimization purposes, except that Darwin’s natural selection is an agentless one, which accounts for the spontaneous interaction of individuals within their species, between, and with their environment (Ruse, 2009).

*On the origin of species by means of natural selection* (Darwin, 1859) contains the basis of Darwin’s theory of evolution, but does not address the issue of Human evolution, to which Darwin devoted two subsequent works: *The descent of Man and selection in relation to sex* (Darwin, 1871a,b) and *The expression of emotions in humans and animals* (Darwin, 1872).

## Genealogy

“[...] the theories of Darwin, which were then of topical interest, strongly attracted me, for they held out hopes of an extraordinary advance in our understanding of the world [...].” (Freud, 1925a, p. 8)

Sigmund Freud’s works have often been compared to the scientific input of Charles Darwin. This has been the case including during Freud’s lifetime, by Eugen Bleuler (1857–1939) (Borch-Jacobsen and Shamdasani, 2012) and Ernest Jones (1879–1958) (Jones, 1913, 1918), for instances; this was also the case by a number of authors after Freud’s death (Shakow and Rapaport, 1964). It must be pointed out that, on several occasions, Freud himself had previously compared the scientific contribution of psychoanalysis to that of Darwin (Freud, 1916–1917, 1925b).

“In the course of centuries the naïve self-love of men has had to submit to two major blows at the hands of science. […] This is associated in our minds with the name of Copernicus, though something similar has already been asserted by Alexandrian science. The second blow fell when biological research destroyed man’s supposedly privileged place in creation and proved his descent from the animal kingdom and his ineradicable animal nature. This reevaluation has been accomplished in our own days by Darwin, Wallace and their predecessors, though not without the most violent contemporary opposition. But human megalomania will have suffered its third and most wounding blow from the psychological research of the present time which seeks to prove to the ego that it is not even master in its own house [...].” (Freud, 1916–1917, pp 284–285)

Freud’s proclamation seems quite draconian. In fact, scientific affiliation to Darwin was nothing very original at this time (Assoun, 1981; Borch-Jacobsen and Shamdasani, 2012), and was if anything a common “genealogical schema” (Assoun, 1981), with Darwinism being in great vogue in the German scientific community from the 1870s (Richards, 2013).

The 1870s were the period of Freud’s medical studies in Vienna (1873–1882), during which he took the neuropathology lessons of Theodor Meynert (1833–1892), who frequently cited Darwin’s *The expression of emotions in humans and animals* (Ritvo, 1990), and before that (1874) the course devoted to Darwinism by zoologist Carl Claus (1835–1899), who personally knew Darwin (Ritvo, 1970). It is also known that Freud bought between 1875 and 1883 the German editions of Darwin’s main works (eight in all, including those of *On the origin of species by means of natural selection, The descent of Man and selection in relation to sex*, and *The expression of emotions in humans and animals* (Ritvo, 1990), and that he read from this time several Darwinian authors, including German biologists Ernst Haeckel (1834–1919) and August Weismann (1834–1914), as well as American philosopher James Baldwin (1861–1934) (Jones, 1957; Ritvo, 1990).

## Emotions

“[...] it occurred to me that the insane ought to be studied, as they are liable to the strongest passions, and give uncontrolled vent to them.” (Darwin, 1872, p. 13)

In *The expression of emotions in humans and animals*, Darwin aimed at demonstrating that emotions and their expressions had a universal nature in Humans and other animals, and that they depended on inherited reflexes – knowing, as we already mentioned, that for Darwin inheritance could concern certain acquired characteristics. This scrupulous work rested upon a great variety of observations – including ones gathered from psychiatrists (Marcaggi and Guénolé, 2018) – and led to Darwin’s formulation of three general principles governing emotional expression (Darwin, 1872, p. 28 and following).

The first one was the principle of “serviceable associated habits,” which accounted for the associations between an emotional experience, what triggered it, the behavioral expression of this emotion, and its immediate consequences: when the behavioral expression of an emotion has had positive consequences, this behavior is automatized as a response to what initially triggered it. For Darwin, this first principle was at the basis of inherited behavioral expressions of emotions, as for example with the Dog when he adopts a posture of attack in front of another one: he stiffens, his tail rises, his hair bristles and his gaze is fixed.

The second principle was “antithesis” (Darwin, 1872, p. 28 and p. 50 and following): when an emotional context is the opposite of another one having itself generated a “serviceable habit,” the individual expresses his emotion with a behavior representing the opposite; for example, the dog adopts regarding his master a posture signifying the opposite to the attack one: he kowtows to him, relaxes his muscles, and his hair becomes smooth. For Darwin, this second principle had a communicational purpose, and could also generate inherited predispositions in siblings.

Finally, the third principle was the existence of “actions due to the constitution of the nervous system, independently from the first of the will, and independent to a certain extent of habit” (Darwin, 1872; p. 28 and p. 66 and following), which encompasses physiological changes spontaneously associated with emotions which we nowadays relate to activation of the autonomic nervous system (tremors or acceleration of heart rate, for examples).

According to Darwin, combining these three principles allowed understanding most aspects of emotional expression in Man and other animals, and Freud made use of them from the beginning of his works on neuroses. Indeed, Freud’s 16 references to Darwin in his official psychoanalytical works (those published in his lifetime) start with two mentions of *The expression of emotions in humans and animals* in *Studies on hysteria* (Freud and Breuer, 1895), where he actually used Darwin’s three principles of emotional expression, in order to explain some hysterical symptoms (Ritvo, 1990). He especially used Darwin’s idea of a dispersion in the body of emotional excess, by “overflow of nerve-force” (Darwin, 1872, p. 76), which might thus be counted in the development of the concept of hysterical conversion (Sulloway, 1979):

“Some of the striking motor phenomena exhibited by Frau von N. were simply an expression of the emotions and could easily be recognized in that light. Thus, the way in which she stretched her hands in front of her with her fingers spread out and crooked expressed horror, and similarly her facial play. [...] Others of her motor symptoms were, according to herself, directly related to her pains. She played restlessly with her fingers (1888) or rubbed her hands against one another (1889) so as to prevent herself from screaming. This reason reminds one forcibly of one of the principles laid down by Darwin to explain the expression of emotions – the principle of overflow of emotions, which accounts, for instance, for dogs wagging their tails.” (Freud and Breuer, 1895, p. 91)

Through his description of the mechanism of natural selection of individuals, Darwin had put two instincts at the heart of life’s evolution: the instinct of self-preservation and the sexual instinct, with an emphasis on the second one. Like many of his contemporaries in the field of psychological medicine (Ellenberger, 1970), Freud thought sexuality had an important role in pathogenesis (Freud, 1905) and, moreover, his first drive theory, elaborated from *Three essays on sexuality* (Freud, 1905) and fully exposed 10 years later in *Instincts and their vicissitudes* (Freud, 1915), established a dualism of ego/sexual drives which, according to Freud himself (Freud, 1913), corresponded to the two instincts mentioned above. More generally, it can be said that Freudian psychopathology before the First World War espoused the ambient Darwinism, as it put an accent on instinct and emotions, and thus on the non-rational aspects of the human psyche.

## Overview

“When the constitutional factor of fixation comes into consideration, acquisition is not eliminated thereby; it only moves into still earlier prehistory, because one can justifiably claim that the inherited dispositions are residues of the acquisition of our ancestors.” (Freud, 1915/1987, p. 7)

Freud’s Darwinian streak went further. As early as 1895, he suggested that certain hysterical symptoms were vestiges of formerly functional instinctive behaviors:

“All these sensations and innervations belong to the field of ‘The expression of emotions,’ which, as Darwin has taught us, consist of actions which originally had a meaning and served a purpose [...]” (Freud and Breuer, 1895, p. 181).

According to several authors (Ritvo, 1990; Laplanche, 2006), this was reinforced in Freud’s thought after he abandoned the idea of a psychotraumatic etiology of hysteria (Ritvo, 1990), and he will later suggest that hysteria, as well as certain phobias, were emotional residues from the prehistory of the human species (Freud, 1916–1917). The hypothesis of a hereditary transmission of certain emotional complexes also figures in *Totem and taboo* (Freud, 1913), where it is very prudently ventured as one of perpetuating factors for parricide and incest prohibitions in human societies since the primal hords conceived by Darwin (Darwin, 1871a):

“The barrier against incest is probably among the historical acquisitions of mankind, and, like other moral taboos, has no doubt already become established in many persons by organic inheritance (Cf. my *Totem and Taboo*, 1912–1913.).” (Freud, 1905, p. 225; added in 1915)

Actually, the heart of Freud’s beliefs on these issues, his “historical-Darwinian point of view” (Sulloway, 1979), is probably to be searched in a text he wrote at the beginning of the First World War, and which he never published: *Overview of the transference neuroses* (Freud, 1915/1987). In this short essay, Freud imagined the prehistoric appearance of neuroses by combining his psycho-sexual theory with an idea ventured by Sándor Ferenczi (1873–1933) (Ferenczi, 1913/1970): the last glaciation at the end of the Pleistocene (110,000–10,000 years BCE) would have caused environmental changes for humans, which psychological consequences would have been transmitted since:

“If we pursue Ferenczi’s idea, the temptation is very great to recognize in the three dispositions to anxiety hysteria, conversion hysteria, and obsessional neurosis regressions to phases that the whole human race had to go through at some time from the beginning to the end of the Ice Age […].” (Freud, 1915/1987, p. 11)

Thus, for Freud, the hostility of the glacial environment may have first rendered the whole humanity anxious, and moreover forced humans to a relative sexual frustration, both predisposing them to phobias; secondly, accumulated sexual frustration led to derivative emotional discharges, particularly in women, which was the origin of the predisposition to hysteria. Freud then explained the appearance of obsessional neurosis and finally of “narcissistic psychoneuroses” (dementia praecox, paranoia, melancholy-mania) by later social influences, ranging from the necessity of instinctual repression again to different consequences of patriarchal domination:

“To summarize, we can say: if the dispositions to the three transference neuroses were acquired in the struggle with the exigencies of the Ice Age, then the fixations that underlie the narcissistic neuroses originate from the oppression by the father, who after the end of the Ice Age assumes, continues its role, as it were, against the second generation.” (Freud, 1915/1987, p. 20)

It is actually very difficult to summarize this extravagant text, of which Freud recognized the inconsistencies and the excessive speculations. This is probably what made him abandon its publication, but it seems that he never gave up on the idea that the pathogenic sexuality of Man originated from a dramatic change in his ancestral environment (de Block, 2006).

## A “Great Biological Vision”

“[...] psychoanalysis could claim a high place among the sciences which are concerned with the reconstruction of the earliest and most obscure periods of the beginnings of the human race.” (Freud, 1899, p. 548; added in 1919)

Some authors have considered that Freud espoused an evolutionism that had since expired, based not on the mechanism of natural selection but on inheritance of acquired characteristics, thus a faulty neo-Lamarckism (Jones, 1957; Stevens and Price, 1996). In reality, as we already mentioned, the principle of inheritance of acquired characteristics, present in Lamarck as a condition of his transformism (Corsi, 1992), was also in Darwin’s works and, challenged at the beginning of the 20th century, was abandoned during the interwar period only (Bowler, 1989).

What Darwin refused in Lamarck’s theory (letter to Joseph Hooker on January 11, 1844; Darwin, 1987) was mostly the idea that biological needs, through the repeated efforts that they led individuals to do, could cause qualitative anatomical modifications, such as the appearance of a new organ, or a structural modification of an already existing organ. However, Darwin supposed that some reflex behaviors initially acquired under the effect of an emotion could be transmitted to the following generations, as for example the flight of certain animals in front of Man (Darwin, 1871a).

For Darwin, and for Freud, Lamarckism was thus an evolutionism centered on a progressive physiological transformation of individuals by repetition of actions born of needs, and it is true that Freud had a period of great enthusiasm about the work of Lamarck, at the end of 1916 and the beginning of 1917, when he read Lamarck’s *Philosophie zoologique* (Jones, 1957; Ritvo, 1990) – probably through his frequent interactions with Ferenczi at this time.

As suggested in a letter to Ferenczi on January 25, 1917 [90], it was finally the “psycho-Lamarckism” of German zoologist and philosopher August Pauly (1850–1914) which most elicited Freud’s theoretical enthusiasm: needs would not transform individuals only trough repeated actions, but would more again act directly on the body (Pauly, 1905):

“I have finally received some books on Lamarck. My impression is that we are coming completely into line with the psycho-Lamarckists, such as Pauly, and will have little to say that is completely new [...]” (letter to Sandor Ferenczi on January 28, 1917; Krutzen, 2005).

Arguing against Darwin’s theory of natural selection, Pauly had claimed (Pauly, 1905) that evolutionary change came from a kind of primordial consciousness, inherent to the very nature of all biological forms and acting spontaneously for self-adaptive purposes, which thus involved needs or psychic desires for its organic realization – hence the was qualified as a psycho-Lamarckian (Cuénot and Tétry, 1951).

Freud’s wish, his “great biological vision” (Jones, 1957, p. 335), was that psychoanalysis, through psycho-Lamarckism, could bring an ultimate understanding of evolution:

“Our intention is to base Lamarck’s ideas entirely on our own theories and to show that his concept of “need” which creates and modifies the organs is nothing other than the power of unconscious ideas on the body [...]” (letter to Karl Abraham on November 11, 1915; Krutzen, 2005).

## Ontogeny and Phylogeny

“The germ cell of a living animal is obliged to repeat in its development – although in a fleeting and curtailed fashion – the structures of all the forms from which the animal is descended […].” (Freud, 1920, p. 308)

Though Freud yet seemed to adopt a neo-Lamarckian vision of evolution since the period of the First World War, it must be added that he was merely and above all a “recapitulationist”: an adept of the “basic biogenetic law” (Haeckel, 1870, p. 361) formulated by Darwin’s great German popularizer, the distinguished biologist from Iena Ernst Haeckel (1834–1919).

This “law,” the most general one among all those described by Haeckel, a bold and prolific theorician, stated in summary and in terms invented by himself: “ontogeny is a short and fast recapitulation of phylogeny” (Haeckel, 1866, p. 300). This idea was in fact an old one (Gould, 1977), and also extended an opinion ventured by Darwin but with which he never dealt in depth (Richards, 1992).

Schematically, the Haeckelian theory of recapitulation postulated (Haeckel, 1866): (1) the acquisition by certain living beings of new adaptive characteristics at the end of their development, that is, in adulthood; (2) the transmission of these acquired characteristics to the descent, with a terminal appearance in the development; and (3) an acceleration of ontogenesis maintaining the duration of development over generations. This “basic law” summarized a broad embryological view, yet Haeckel never gave it a real predictive value, given the many exceptions that it suffered (Gliboff, 2008; Richards, 2008). However, by its apparent obviousness, “ontogeny resumes phylogeny” turned out to be an irresistible slogan: it prevailed in minds with its full simplicity and all the biologists of the late XIX^TH^ and early XX^TH^ centuries embraced recapitulationism, whatever they were Darwinian or not (Gould, 1977).

It was so with Freud, who mobilized the basic biogenetic law in his writings on nearly twenty occasions (Delrieu, 2008), from 1910, including in Haeckel’s own words:

“Ontogenesis may be regarded as a recapitulation of phylogenesis, in so far as the latter has not been modified by more recent experience [...].” (Freud, 1905, p. 131; added in 1915)

“Behind this childhood of the individual we are promised a picture of a phylogenetic childhood—a picture of the development of the human race, of which the individual’s development is in fact an abbreviated recapitulation influenced by the chance circumstances of life […].” (Freud, 1899, p. 548; added in 1919)

“[…] each individual somehow recapitulated in an abbreviated form the entire development of the human race […].” (Freud, 1916–1917, p. 199)

Freud had presumably studied Haeckel at the beginning of his medical studies, since he advised his reading at that time to one of his close friends (letter to Eduard Silberstein on 20 December, 1874; Krutzen, 2005); he however never mentioned the name of Haeckel himself in his writings, only that of his great popularizer and biographer Wilhelm Bölsche (1861–1939) (Amouroux, 2004).

Whatever it may be, Freud really conceptualized the evolution of the human psyche using a strong and literal version of the basic biogenetic law, combined with anthropological evolutionism of this time (Sanderson, 1990); for Freud, the acquisition of walking, the Oedipus complex, and the latency period repeated the prehistory and history of Man from his acquisition of vertical posture to appearance of monotheistic civilization, through totemism and the prohibition of incest (Duvernay Bolens, 2001). This constituted a massive recapitulationism, far exceeding the conceptions of Haeckel himself. In this context, Freud seemed to consider mental disorders, at first neuroses, as ontogenetic fixations-regressions informative about the prehistory of humanity.

## Freudian Cost

“My position, no doubt, is made more difficult by the present attitude of biological science which refuses to hear of the inheritance of acquired characters by succeeding generations. I must, however, in all modesty confess that nevertheless I cannot do without this factor in biological evolution. […] If this is not so, we shall not advance a step further along the path we entered on, either in analysis or in group psychology.” (Freud, 1939, pp 99–100)

The theory of recapitulation and the principle of inheritance of acquired characteristics were challenged from the end of the First World War, with the development of experimental embryology and the recognition of the principles of Mendelian genetics, and both gradually abandoned in the interwar period (Gould, 1977).

Although Freud has always been cautious about these issues in his published work, a number of sources attest to his attachment to these outdated biological principles until the end of his life (Jones, 1957; Laplanche, 2006). One can thus wonder of course about what would have changed in Freud’s works a renunciation in the interwar period of psycho-Lamarckism, the law of recapitulation, and more broadly the notion of inheritance of acquired characteristics.

Answering these “what if” questions in detail would probably represent a long-term work of exegesis and “counterfactual history” (Bunzl, 2004), and we thus do not claim here to revisit the whole of Freud’s works from this angle. Nevertheless, we propose to sketch out the main lines of this inventory, distinguishing between what would have amounted to an abandonment of each one of the three notions mentioned.

Even if Freud was strongly interested in psycho-Lamarckism – i.e., the evolutionary theory according to which species adapt over generations by the effect on the body of individual will and resulting actions – and even enthusiastic regarding its theoretical perspectives at the end of the First World War, it does not in itself constitute a necessary postulate in the rest of his work.

Haeckel’s “law of recapitulation” seems to have been for Freud a lasting and significant source of inspiration, especially to conceive the innate aspects of his theory of childhood psychosexual development. This corresponds to what Laplanche described as Freud’s “misleading biologizing of sexuality” (Laplanche, 2006), and renouncing it probably would have led Freud if not to rework his conception of infantile sexuality, at least to give it more social and cultural interpretations.

The renunciation of the principle of inheritance of acquired characteristics would also have led Freud to reconsider the role and mechanisms of intergenerational and cultural transmission in the anthropological aspects of his works. Nowadays, “psychoanalytic anthropology” (Paul, 1989) has moreover to integrate the neo-Darwinian conceptions of evolution to its understanding of the prehistory of the human unconscious. For example, Paul proposed a hypothesis regarding the emergence and intergenerational transmission of the taboo of incest and the Oedipus complex, which combines the different evolutionary mechanisms composing the “extended synthesis” (Pigliucci and Müller, 2010): individual natural selection, niche construction (gene × culture coevolution), group selection, and the Baldwin effect (Paul, 2010).

According to this hypothesis, the nuclear family unit, which succeeded 100s of 1000s of years ago to male dominance hierarchy within large human groups (protohuman societies), was brought into existence by generalized rebellions of peripheral males against dominant ones (Paul, 2010). These rebellions were made possible by a prior natural group selection of human nomadic hunter-gatherers who had developed social cooperation by gene × culture coevolution, and previously the language/symbolic cognition and reversibility of thought which it allowed (Knight et al., 2000). Paul also hypothesizes a subsequent Baldwin effect: though much more egalitarian than protohuman societies, human societies after generalized “primal crimes” still included male dominance hierarchy within family units, and natural selection could have operated for male individuals who had highest propensity to learn and accept a new taboo of incest. This “just so story” (Gould) both includes ingredients of Freud’s “historical-Darwinian point of view” (Sulloway, 1979) and is in keeping with current evolutionary thought and knowledge regarding human history (Todd, 2017).

The current interest for epigenetic inheritance (Gissis and Jablonka, 2011) has sparked renewed interest in Freud’s assumptions in transmission of traumatic memory across generations (Fischmann, 2016). However, it is important to remain very cautious on this subject at the moment; indeed, though epigenesis – i.e., changes in gene function that do not involve changes in the DNA sequence – is a well-demonstrated phenomenon with clear developmental implications at the individual level, involvement of epigenetic mechanisms in intergenerational transmission has been yet little documented in humans (Yehuda et al., 2016), and never across several generations. Scientific conclusions in this area thus remain fragile for the moment, and it seems in any case very unlikely that epigenesis could account for intergenerational transmission of unconscious mnemonic contents or predispositions for complex behavioral patterns.

Whatever it be, the fact remains that Freud was probably the first to elaborate on “ultimate” psychological causes of mental syndromes, not only “proximate” ones – to use terms coined by biologist Nikolaas Tinbergen (1907–1988) – which indeed makes of him the main forerunner of current evolutionary psychopathology (Stevens and Price, 1996; McGuire and Troisi, 1998; de Block, 2006). At the end of his career, he expressed the wish that studying evolution would be systematically part of the training of psychoanalysts (Freud, 1926).

## Conclusion: Laughing Rats

“In the beginning was emotion.” (Céline, 1958; A)

Two close collaborators of Freud also made psychopathological contributions involving evolutionary concepts: Carl-Gustav Jung (1875–1961), with his notion of a “collective unconscious” (Jung, 1916/1981); and of course Sándor Ferenczi, whose essay *Thalassa* explicitly pursued the psycho-Lamarckian speculations initiated with Freud (Ferenczi, 1924/1974). However, both did not directly draw inspiration from Darwin, nor did the major psychoanalysts of the first half of the 20th century – except maybe Heinz Hartmann (1894–1970), with his focus on an adaptative *ego* (Hartmann, 1939).

The return of a Darwinian inspiration in psychopathology actually occurred in the era of “neo-Darwinism,” the synthetic theory of evolution developed from the 1930 to 1950s, by combining the Darwinian principle of natural selection with the contributions of genetics and paleontology (Bowler, 1989). It was through contact with one actor of this synthesis, the biologist Julian Huxley (1887–1975), in the 1950s that John Bowlby (1907–1990), then a young psychoanalyst, took an interest in ethology and evolutionary hypotheses about mother–infant bonding (Bowlby, 1969) – and created in the process the notion of an “environment of evolutionary adaptedness,” supposed to designate the African and then Eurasian Pleistocene environment within which human hords were potentially subject to natural selection.

Though Bowlby’s seminal works stay relevant for a great part, the neo-Darwinian theorization of the whole social behavior through sociobiology (Wilson, 1975), and currently through evolutionary psychology – its cognitivist continuation (Tooby and Cosmides, 1995) – has produced what we would call a “generalized adaptationism,” which is open to serious criticism for begging many questions (Gould and Lewontin, 1979; Fodor, 1998) and for its tendentiously conservative preconceptions (Lewontin et al., 1984; MacKinnon, 2005). The resulting neo-Darwinian current in psychopathology and psychiatry (Stevens and Price, 1996; Baron-Cohen, 1997; McGuire and Troisi, 1998; Brüne et al., 2012) thus professes an adaptationist modelization of mental disorders, by postulating as ultimate causes genetic predispositions formerly advantageous in the “environment of evolutionary adaptedness,” for the individual and/or the group.

Without rejecting this scientific ambition, we can note that evolutionary psychopathology suffers in its present form from certain major weaknesses. In particular, its works almost only focus on human evolution, and thus very little take into consideration prehuman evolution, particularly the one of emotional systems of the reptilian-paleomammalian brain since the appearance of vertebrates (Panksepp and Panksepp, 2000). More globally, modular hypotheses in the field of current evolutionary psychopathology rest solely on behavioral and cognitive bases, and thereby include no neuroscientific corroborations or demonstrated correlates (Ellis and Solms, 2018).

Indeed, a truly Darwinian approach to human mind and behavior must necessarily take into account the whole neurological and psychological evolution of species (Panksepp and Biven, 2012), as it has been the case in the field of affective neuroscience (Panksepp, 1998). Within this framework, mental disorders are seen primarily as diverse disturbances involving the basic emotional systems of the human brain (Panksepp, 2006); an approach which is consistent with the main current developments in philosophy of psychiatry (Wakefield, 2007). As was recently described by Ellis and Solms, basic emotional systems seem to constitute much more robust candidates to the status of real psychological modules than the myriad of domain-specific cognitive systems postulated by evolutionary psychologists (Ellis and Solms, 2018).

*From an evolutionary point of view*, emotions and the neural mechanisms underlying their expressions represent the bedrock of the psyche, and play a key role in the development of individuals as their first mode of apprehending the external world (Solms and Turnbull, 2002). As a long-established science of emotions and their vicissitudes in human beings, psychoanalysis may thus still have a role to play for a rational Darwinian approach to psychopathology.

## Author Contributions

GM wrote the first draft of the manuscript. FG wrote sections of the manuscript. Both authors contributed to manuscript revision, read and approved the submitted version; contributed conception and design of the article.

## Conflict of Interest Statement

The authors declare that the research was conducted in the absence of any commercial or financial relationships that could be construed as a potential conflict of interest.

## Footnotes

1 It can no longer be said, however, that the principle of inheritance of acquired characteristics is, strictly speaking, obsolete at present. After having been there for nearly a 100 years, it now comes back to the heart of the concerns of behavioral genetic researchers, through the field of intergenerational epigenetic transmission, on which we will return in more details later in the article (see section “Freudian Cost,” penultimate paragraph).
